# Antibiotic Prescription in Veterinary Consultations in Bhutan: A Retrospective Cross-Sectional Study

**DOI:** 10.3389/fvets.2021.641488

**Published:** 2021-05-28

**Authors:** Juan Pablo Villanueva-Cabezas, Karma Rinzin, Sithar Dorjee, Pema Tshewang, Ugyen Namgyel, Puspa Maya Sharma, Mark A. Stevenson, Jodie McVernon

**Affiliations:** ^1^Department of Infectious Diseases, Peter Doherty Institute for Infection and Immunity, University of Melbourne, Melbourne, VIC, Australia; ^2^Animal Health Division, Department of Livestock, Thimphu, Bhutan; ^3^Department of Epidemiology, Khesar Gyalpo University of Medical Sciences, Thimphu, Bhutan; ^4^National Veterinary Hospital, Department of Livestock, Thimphu, Bhutan; ^5^National Centre for Animal Health, Department of Livestock, Thimphu, Bhutan; ^6^Melbourne Veterinary School, Faculty of Veterinary and Agricultural Sciences, The University of Melbourne, Melbourne, VIC, Australia

**Keywords:** antibiotic prescription, veterinary antibiotic, antibiotic stewardship, antibiotic treatment, antibiotic resistance, veterinary service, One Health, low-and middle-income countries

## Abstract

The veterinary prescription of antibiotics in low- and middle-income countries (LMIC) remains largely undocumented. In Bhutan, however, the national veterinary service keeps records of their activities and prescriptions, which offer an opportunity to establish a benchmark to assess the use of these agents in this and other LMIC. A cross-sectional retrospective study was designed and 2,266 handwritten veterinary records from 2017 were sampled from 23 animal health premises (AHPs) to estimate individual and an overall proportion of consultations that resulted in an antibiotic prescription. The frequency of antibiotic prescription per species, type of AHP, and according to WHO's AWaRe index and OIE list of priority antimicrobials were also explored. It was estimated that 31% (95% confidence interval: 29–33%; intracluster correlation: 0.03) of the veterinary consultations resulted in an antibiotic prescription. The incidence of antibiotic prescription was highest in consultations of poultry across AHP. Across species, *diarrhea and wounds* were frequently treated with broad-spectrum antibiotics including sulfonamides, tetracyclines, trimethoprim + sulfa, and penicillin. Between 45% and 70% antibiotics prescribed correspond to AWaRe's *access group* and up to 25% to AWaRe's *watch group*. Over 70% of antibiotics dispensed in veterinary consultations for any species correspond to the OIE's *veterinary critically important antimicrobial agents*. Overall, the study demonstrated positive features of veterinary antimicrobial stewardship in Bhutan, given the conservative proportion of consultation that results in this type of prescription and the type of antibiotic prescribed. Although the veterinary service closely follows the Bhutanese Standard Treatment Guidelines, the prescription of antibiotics to key species should be closely monitored. Our study suggests that further improvements of antibiotic stewardship can be achieved through standardisation of antibiotic prescription to some species, a revision of the guidelines toward reducing the prescription of antibiotics of high relevance for human medicine, and by including details of clinical investigation, use of tests, and treatment outcomes in veterinary consultation records.

## Introduction

Antimicrobial resistance (AMR) occurs when microorganisms such as bacteria, viruses, parasites, or fungi change in ways that make the drugs used to treat them ineffective. Although most One Health research emphasizes on zoonotic threats (1), AMR is “the quintessential One Health problem” as it collectively threatens humans, animals, plants, and the whole ecosystem (2). The emergence and spread of new resistance mechanisms to antibiotics is particularly alarming, as it undermines the available arsenal to treat common infectious diseases (3).

The monitoring of veterinary antibiotic usage is context specific. In high-income countries, intense control and regulation have resulted in a reduction of antibiotic prescription and improved stewardship. In low- and middle-income countries (LMIC), antibiotics are less regulated, and there is limited reporting on the extent of their use (4, 5). According to the World Organization for Animal Health (OIE), most LMIC members lack mechanisms to accurately estimate the use of antibiotics in animals, which limits their capacity to report type, dose, route, and purpose of antibiotic dispensing to animals (6). The accelerated intensification of livestock production systems in LMICs, driven by a growing demand for animal-sourced nutrients, is forecast to boost veterinary prescription and dispensing of antibiotics (4). Thus, the establishment of a benchmark to assess veterinary antibiotic usage in LMICs is of growing importance.

Bhutan, a landlocked nation in the Himalayas, has unique features of livestock management and veterinary health care delivery among the LMICs. According to Bhutanese principles, animals are rarely slaughtered and instead are cared for until they die of natural causes. In Bhutan, the veterinary service is public and free of charge; veterinarians and para-veterinarians follow the Standard Treatment Guidelines of Bhutan (7) compiled by the National Veterinary Hospital in 2017 to standardise diagnosis and prescription across the country and keep handwritten records of their activities. Although Bhutan has worked toward strong institutional antimicrobial stewardship, the “[.]lack of knowledge on the use and consumption of antimicrobial agents across One Health sectors” remains a crucial gap in antimicrobial resistance surveillance (8). The examination of past veterinary consultation records can help to address this knowledge gap and provides a benchmark to assess interventions to promote veterinary antibiotic stewardship in this country.

Thus, this research aimed to conduct a retrospective study of veterinary consultation records to primarily estimate the proportion of veterinary consultations that resulted in antibiotic prescription in Bhutan in 2017. The study described veterinary antibiotic usage in relation to both WHO's *AWaRe index* for antibiotic stewardship (9), and the OIE's list of antimicrobial agents of veterinary importance (10).

## Methods

This study was approved by the Human Ethics Advisory Group at the Faculty of Veterinary and Agricultural Sciences, The University of Melbourne (Ethics ID: 1853519.1). The study is reported according to the STROBE-Vet statement (11).

### Study Area and Study Design

The Kingdom of Bhutan is administratively divided into 20 *Dzongkhags* (districts) which are further divided into 205 *geogs* (groups of villages). In Bhutan, the veterinary service has a hierarchical structure. Livestock and companion animals in each geog receive health care from personnel employed in two types of animal health premises (AHP): para-veterinarians in local Livestock Extension Centers (LEC) or veterinarians employed by the Dzongkhag Veterinary Hospital (DVH). In geogs where a DVH exists, the LEC is incorporated into the DVH. Beyond their local dispensing of health care, the veterinarians and laboratory personnel of the DVH oversee and provide technical support to all LEC under their Dzonghkag jurisdiction. DVHs, in turn, receive technical support from one of four Regional Livestock Development Centers (RLDC), and these from the National Center for Animal Health (NCAH).

To estimate the proportion of veterinary consultations that resulted in an antibiotic prescription, we designed a retrospective cross-sectional study of veterinary consultation records (the unit of interest) performed in the West Regional Livestock Development Center jurisdiction of Bhutan in 2017 (WRLDj; Dzongkhags Chhukha, Haa, Paro, Samtse, and Thimphu). We focused on this year because this was the last year for which both consultation records and an official livestock census (12) were available at the time of study design. We purposefully selected the WRLDj because it had the largest livestock population in 2017 (12), was geographically accessible, and allowed for appropriate use of the time, human, and economic resources available. The veterinary consultation records are handwritten reports (in English) kept in official notebooks; when these are complete, they include the date of consultation, address (village), livestock species attended, type of animal (local or crossbreed), sex, age (young or adult) number of animals presented, diagnosis (usually a sign of disease or syndrome), and treatment prescribed.

### Sample Size

We estimated the sample of veterinary records necessary to answer the research question using a combination of expert opinion, preliminary analysis, and a cluster-sample design. Based on preliminary analysis of veterinary records from two LEC and local expert opinion, we estimated that the number of consultations per LEC in 2017 was between 300 and 400, and that about one-third resulted in the prescription of antibiotics. To ensure adequate representation of syndromes and treatments throughout 2017 (the consultations were not equally distributed through the year), it was determined that 120 records (10 random records per month) would be collected from each AHP. To estimate the number of AHP sourcing veterinary records, an intraclass correlation estimate for the antibiotic prescription proportion was needed; however, this estimate is unknown in Bhutan. We assumed a conservative design effect of 5.5 [assumed intraclass correlation (ICC) (5.5–1)/(120–1) = 0.04] to ensure appropriate study power. Thus, if 33.3% of the veterinary consultations in 2017 resulted in an antibiotic prescription and 120 veterinary records were collected from each AHP, then we estimated that 17 AHP and 2,040 veterinary records in total were needed to be 95% confident that our estimate of the proportion of veterinary consultations that result in an antibiotic prescription was within 5% of the true population value [that is, a relative error of 0.05/0.33 = 0.15; Levy and Lemeshow (13), p. 70–75].

To select the AHP sourcing the consultation records, we performed a probability proportional to size sampling (13). We assumed that AHP in geogs with a large livestock population would attract more frequent and diverse veterinary consultations than those located in less populated geogs. To make the geogs' livestock population comparable, we aggregated the species recorded in the livestock census 2017 (equine, swine, poultry, sheep, goat, and cattle) into *Livestock Units* (LSU) following standard methods (14). Dogs and cats were excluded from this estimation as there is no standard approach to aggregate these species. A cluster sampling interval, obtained from dividing the cumulative sum of LSU by the number of AHP to be sampled (i.e., 110,908 divided by 17), was used to identify the AHP sourcing veterinary records.

### Data Collection and Analysis

Selected AHP were visited and consultation records collected in June and July 2019. When the AHP was not accessible, or the records were not available, the AHP was replaced (where possible) by another AHP. An AHP was deemed an adequate replacement if it had a similar number of LSU and a similar representation of livestock species. The veterinary consultation records were transcribed *in situ*, or pictures were taken for later transcription into Excel. The diagnoses were term-normalised using regular expression algorithms. Likewise, antibiotics were term-normalised and classified into the corresponding antibiotic class (15). The proportion of veterinary consultations that resulted in an antibiotic prescription in 2017 was estimated using R's *meta* (16) as the pooled estimate of individual AHP proportions with weights assigned under the fixed-effects model [i.e., premises were weighted by the inverse of its variance (17)]. *Post-hoc* analyses to describe patterns of antibiotic prescription were performed using R's *epiR* (18). Finally, the prescription of antibiotics was classified as per the *AWaRe index*, which is a novel WHO's metric for antibiotic stewardship in public health that draws upon the classification of antibiotics in the WHO Essential Medicines List for optimal use (9, 19). Antibiotics were classified into *access* (broad spectrum agents with low resistance potential) or *watch* (agents of the highest priority for human medicine, and with a relatively high resistance potential) agents. The prescription was also classified according to the OIE's list of antimicrobial agents of veterinary importance. The OIE classification uses a different rationale as it weights antibiotics according to the identified agent's importance by OIE state members, and the agent efficacy to treat serious animal diseases and the lack of therapeutic alternatives. Thus, antibiotics were classified into *veterinary critically important antimicrobial agents* (VCIA; antibiotics deemed essential and with limited therapeutic alternatives) and *highly important antimicrobial agents* (VHIA; agents that are either deemed essential against or have limited therapeutic alternatives).

## Results

### Proportion of Consultations That Result in an Antibiotic Prescription

**Figure 1:**
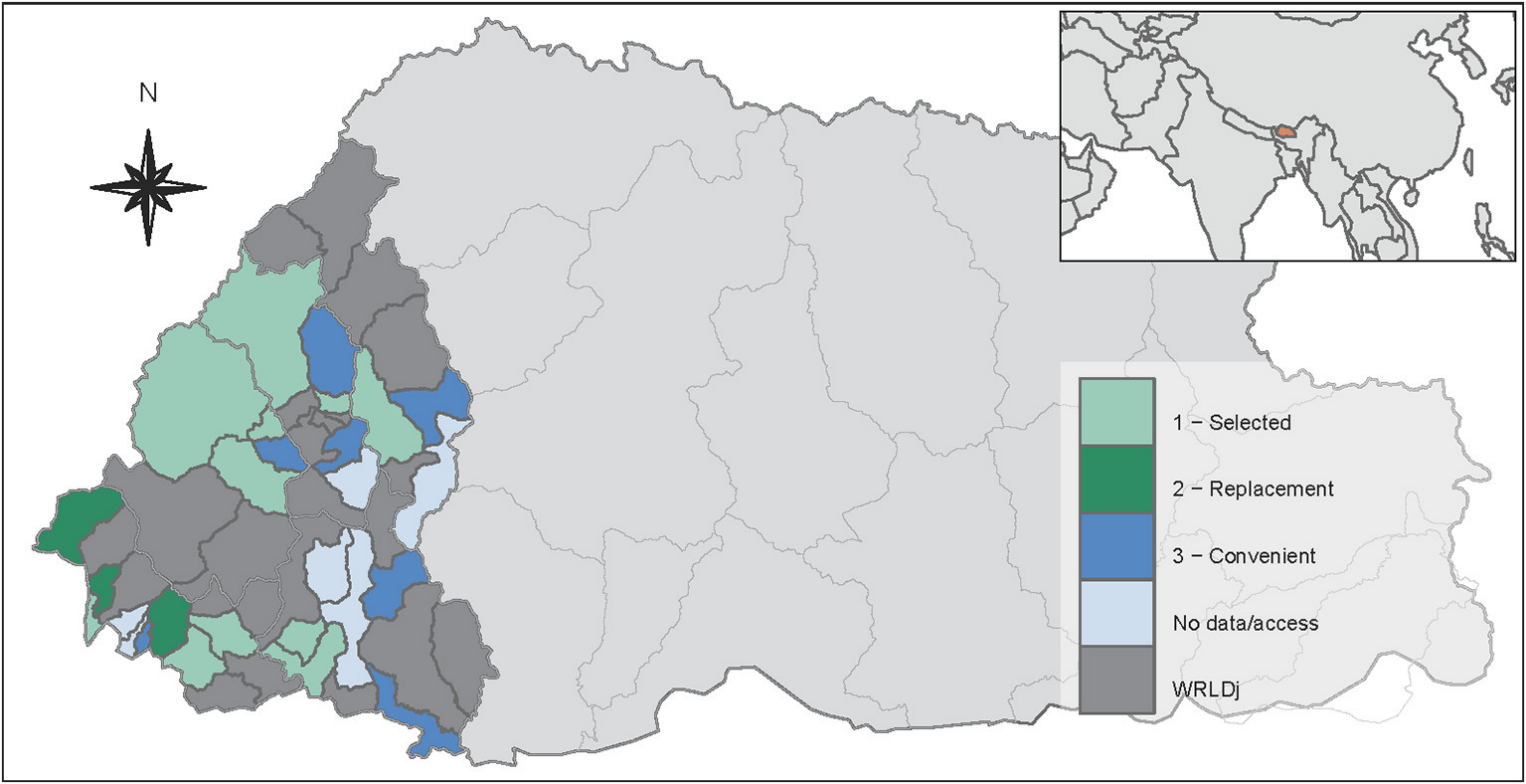
West Regional Livestock Development jurisdiction (WRLDj) of Bhutan. A total of 23 animal health premises (AHP) located in 21 geogs were visited. Eleven AHP were selected and sampled (“selected”), three were chosen as adequate replacements (“replacement”), and another nine were conveniently visited and sampled (“convenient”). A total of 2,266 veterinary consultation records performed in 2017 were collected and scrutinised.

Eleven out of 17 AHP (9 LEC and 2 DVH) initially selected for this study sourced a total of 1,000 veterinary consultation records. Four of the AHPs selected were inaccessible, and two did not keep consultation records for 2017. Other AHPs not considered in the original study design (12 in total) were opportunistically visited and consultation records collected. Three of these AHPs (three LEC) were deemed an adequate replacement for AHPs (three LEC) that could not source data (Figure 1).

**Figure 2:**
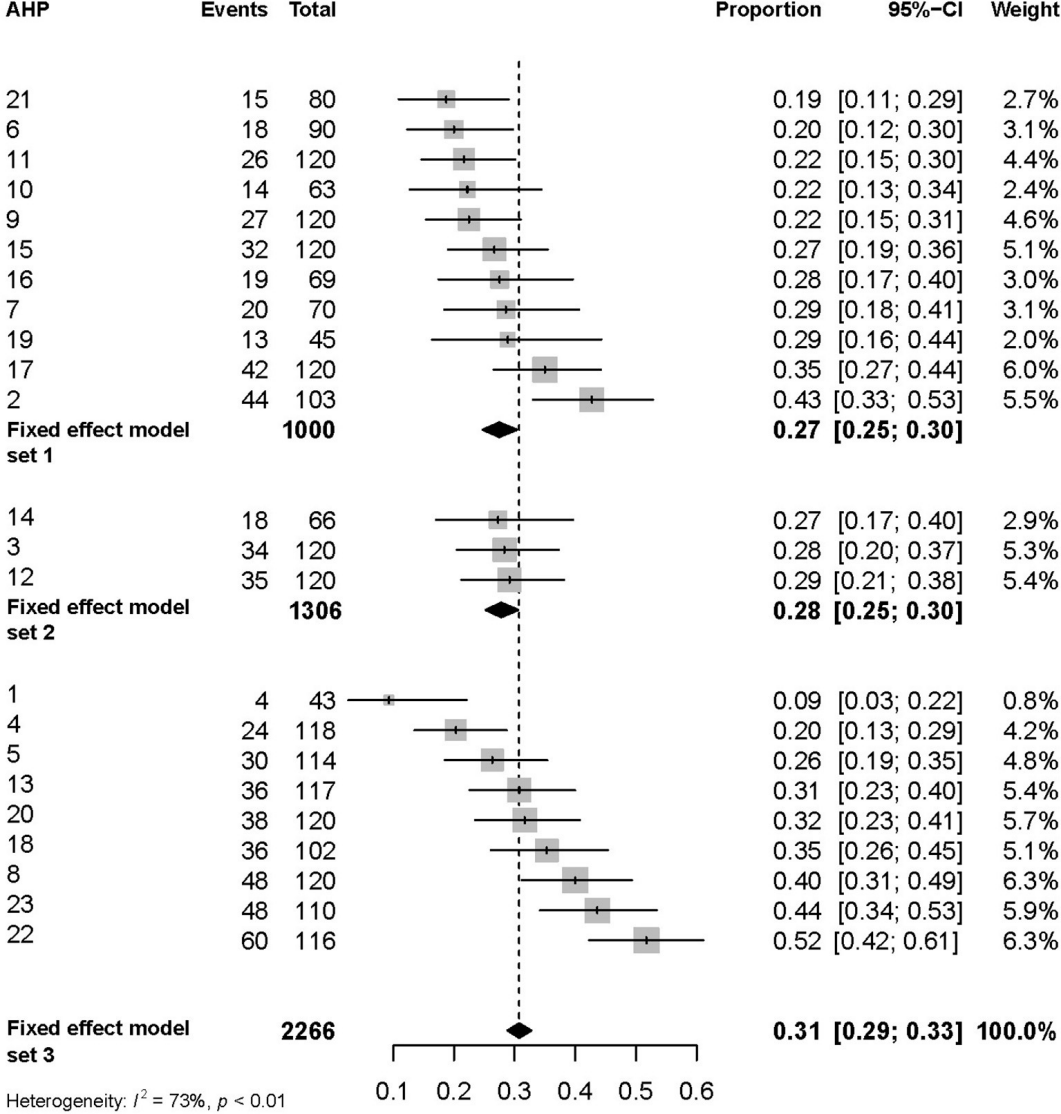
Proportion of veterinary consultations that resulted in an antibiotic prescription in the WRLDj in 2017. Three estimates based on a sequential aggregation of data are reported (see “RESULTS” for details). All estimates suggest that about a third of the veterinary consultations resulted in the prescription of at least one antibiotic (“events”). The global assessment based on set 3 suggests a high level of heterogeneity in the prescription of antibiotics (*I*^2^ = 73%) across the animal health premises (AHP) sampled. AHP were numbered for anonymity.

Accordingly, we report three estimates of the proportion of veterinary consultations that resulted in an antibiotic prescription in 2017 (Figure 2): an estimate based on data sourced by AHPs initially selected for this study (set 1); an estimate based on set 1 plus data sourced by adequate AHP replacements (set 2); and a global estimate based on records sourced by all 23 AHPs visited during the fieldwork (set 3).

The estimate based on set 1 [consultation records *n* = 1,000; median number of consultations per AHP (mAHP-C): 90; interquartile range (IQR): 70–120] suggests that the proportion of veterinary consultations that resulted in antibiotic prescription in 2017 was 27% (95% confidence interval: 25–30%; ICC: 0.017). This proportion ranged between 19% (11–29%) and 43% (33–53%) across AHP. The estimate based on set 2 (*n* = 1,306; mAHP-C: 90; IQR: 68–120) varies minimally (28%; 95% CI: 25–30%; ICC: 0.011), while the estimate based on set 3 (*n* = 2,266; mAHP-C: 114; IQR: 75–120) suggests that 31% (95% CI: 29–33%; ICC: 0.03) veterinary consultations resulted in an antibiotic prescription in 2017.

### Frequency and Distribution of Consultations and Antibiotic Prescription

Characteristic of the veterinary records scrutinised are shown in Table 1. Bovine, canine, and avian (poultry) species are the most frequently mentioned in these records. Bovine, avian, and other livestock consultations are mostly found in LEC's records while canine and feline in DVH's. The median number of animals treated with antibiotics in consultations was generally 1 or 2, although the range is ample. Avian species were usually treated as a flock, with a median of 18 birds in LEC consultations and 235 in DVH's.

**Table 1:** Veterinary consultation records (*n* = 2,266) obtained from 23 animal health premises in WRLDj (set 3).

**Species**	**Records**	**Premise**	**Records**	**AB prescribed**	**Animals AB treated**
			**per premise**		**Median (min–max)**
Bovine	1,498	DVH	338	95	1 (1–20)
		LEC	1,160	300	1 (1–100)
Canine	404	DVH	280	77	1 (1–10)
		LEC	124	34	1 (1–4)
Avian	125	DVH	33	16	235 (1–2,000)
		LEC	92	67	18 (1–1,000)
Equine	71	DVH	5	4	2 (1–12)
		LEC	66	27	1 (1–16)
Caprine	55	DVH	9	5	1 (1–3)
		LEC	46	16	1 (1–5)
Swine	43	DVH	18	5	2 (1–5)
		LEC	25	6	2 (1–10)
Feline	38	DVH	30	18	1 (1–3)
		LEC	8	3	1 (1–1)
Ovine	21	DVH	1	0	–
		LEC	20	5	2 (1–13)
Yak	11	DVH	1	0	–
		LEC	10	3	1 (1–1)

**Figure 3:**
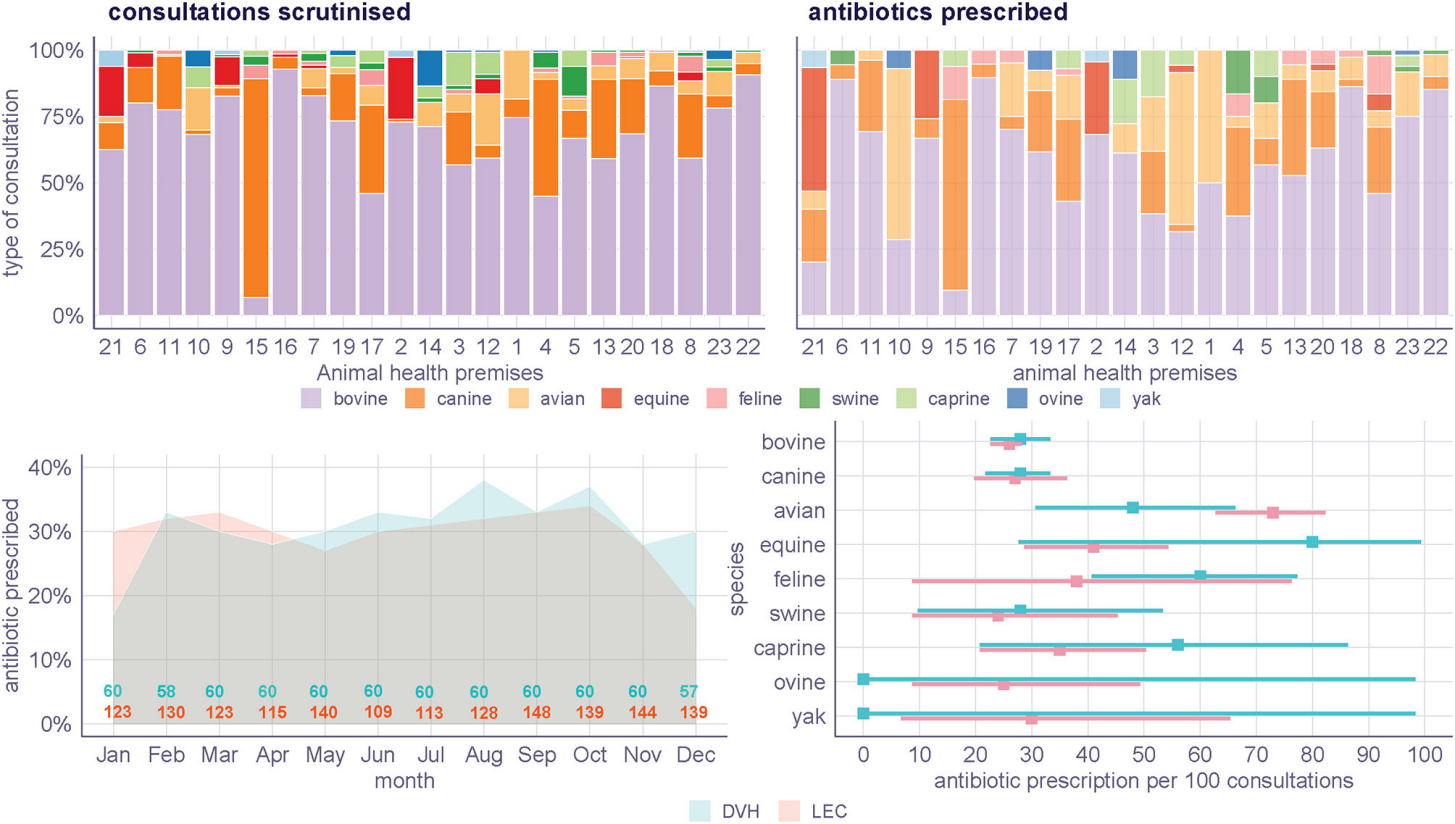
*Post-hoc* analyses. Top, distribution of consultations per AHP (left) and distribution of consultations that resulted in at least one antibiotic prescribed (right, faded). Bottom: distribution of prescription across 2017 and the total number of records that inform each month (left). Incidence of antibiotic prescription (and 95% confidence interval) expressed as the number of veterinary consultations that resulted in an antibiotic prescription per 100 consultations in 2017 (right). LEC, livestock extension center; DVH, district veterinary hospital.

Figure 3 presents the frequency and distribution of consultations and antibiotic prescription. Bovine consultations were predominant across the premises visited, except in AHP 15, where canine were most frequently seen. Other species had a variable representation which was expected based on the livestock census data. Overall, the distribution of consultations with a prescription of antibiotics resembled the distribution of consultation records scrutinised, even in those AHPs that exceeded the regional estimate of antibiotic prescription reported in Figure 2 (AHPs 2, 8, 17, 18, 22, 23). Avian consultations, however, were particularly prone to result in the prescription of antibiotics and represent a large fraction of the total prescription in multiple AHPs (AHPs 10, 12, 1).

The overall proportion of consultations that resulted in antibiotic prescription oscillated around 30% for both DVH and LEC throughout the year 2017. This is explained by a consistent antibiotic prescription incidence in bovine and canine consultations, which represent 84% of all records scrutinised. Among the top 3 species, the incidence of antibiotic prescription in avian consultations is particularly high in LEC with an estimated 75%. The incidence of antibiotic prescription is also high for equine in DVHs, but the limited number of records for this and other species results in significant variability of these estimates.

### Antibiotic Prescription Classified as Per the Aware Index and OIE Classification

**Figure 4:**
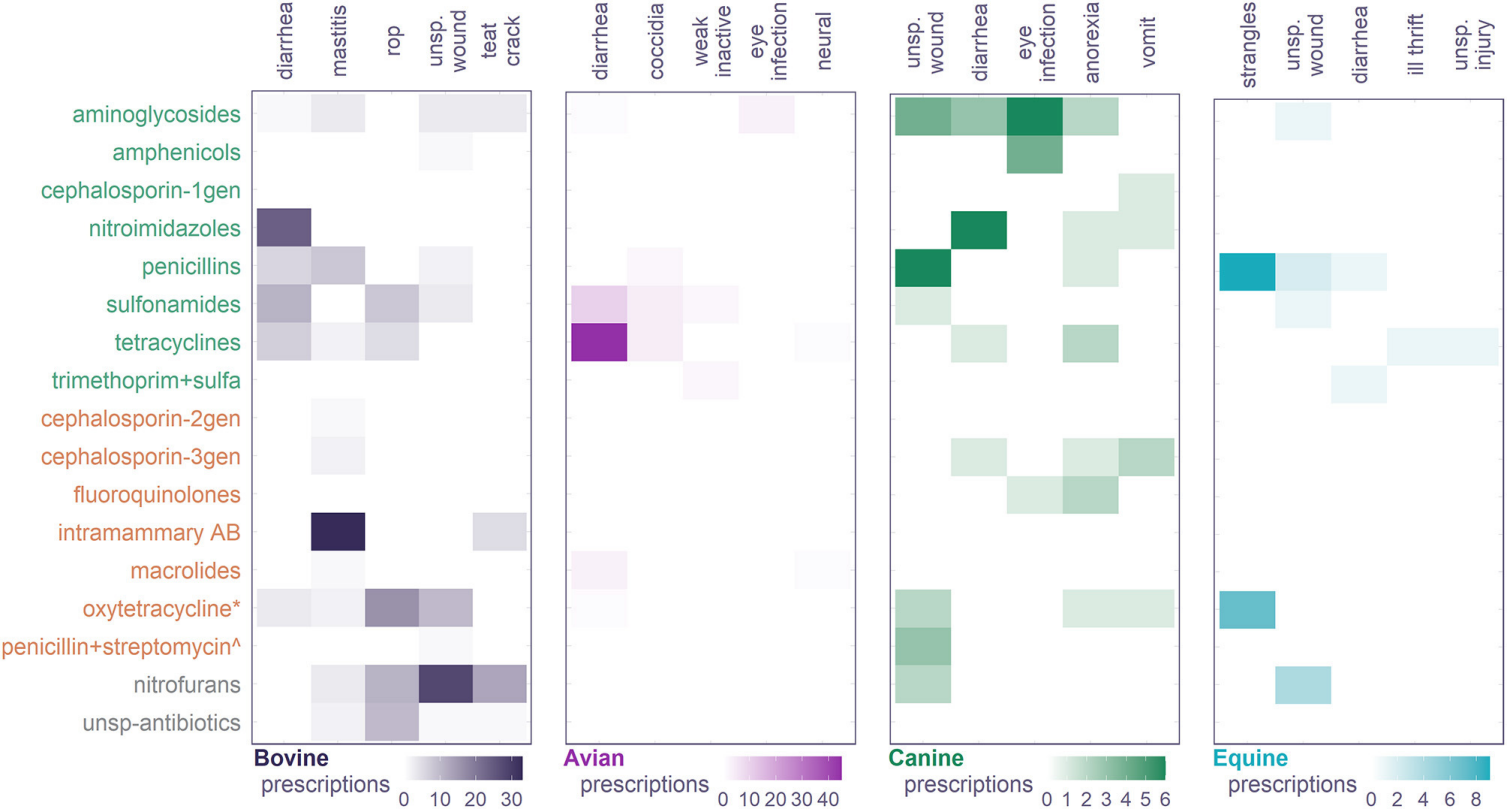
Top 5 diagnosis most frequently treated with antibiotics and associated antibiotic class prescription. Antibiotics classes are colored according to the *AWaRe index*; green: *access*; amber: *watch*; gray: *unclassified*. * and ∧ were separated from their classes as these drugs have different classification. Unsp. wound, unspecific wound; rop, retention of placenta; neural, unspecific neurological signs including lethargy and ataxia; unsp. injury, unspecific injury; miscellaneous, unspecific signs including swollen, stress, and unconnected terms including “preventive” and “no diagnosis”.

Figure 4 presents the five diagnoses most frequently treated with antibiotics. Diarrhea and wounds were frequent health issues across species; diarrhea was usually treated with broad-spectrum antibiotics including nitroimidazoles, sulfonamides, tetracyclines, and trimethoprim (combined with sulfamethoxazole or sulphadiazine); wounds with nitrofuran (nitrofurazone), penicillins, sulfonamides, and tetracyclines (including oxytetracycline). Eye infection was also a top diagnosis for canine, caprine, swine, and feline and was usually treated with aminoglycosides and chloramphenicol. Anorexia was a top diagnosis in three species; six different antibiotics were prescribed to treat it in canine, and two in feline and swine (Figure 4; Supplementary Figure 1).

Most antibiotic classes prescribed in these veterinary consultations were classified as a whole class into AWaRe's *access* or *watch*, with some exceptions: oxytetracycline is classified as a watch antibiotic and not access as the rest of the tetracyclines (19); the formulation penicillin + streptomycin was classified watch given the watch category of streptomycin (19); likewise, “intramammary AB” were classified AWaRe's watch as these formulations include penicillin + streptomycin. Nitrofurans have not been classified into the AWaRe index, and unspecific antibiotics reported in several consultation records could not be classified at all. Figure 4 shows that access agents are the primary prescription option to treat these common diagnoses; the exception was the treatment of mastitis in bovine, which largely falls into AWaRe's watch given the presence of streptomycin in the intramammary tub most frequently prescribed. Oxytetracycline was often used to treat mastitis and other top afflictions of bovine, particularly wounds and retention of placenta.

**Figure 5:**
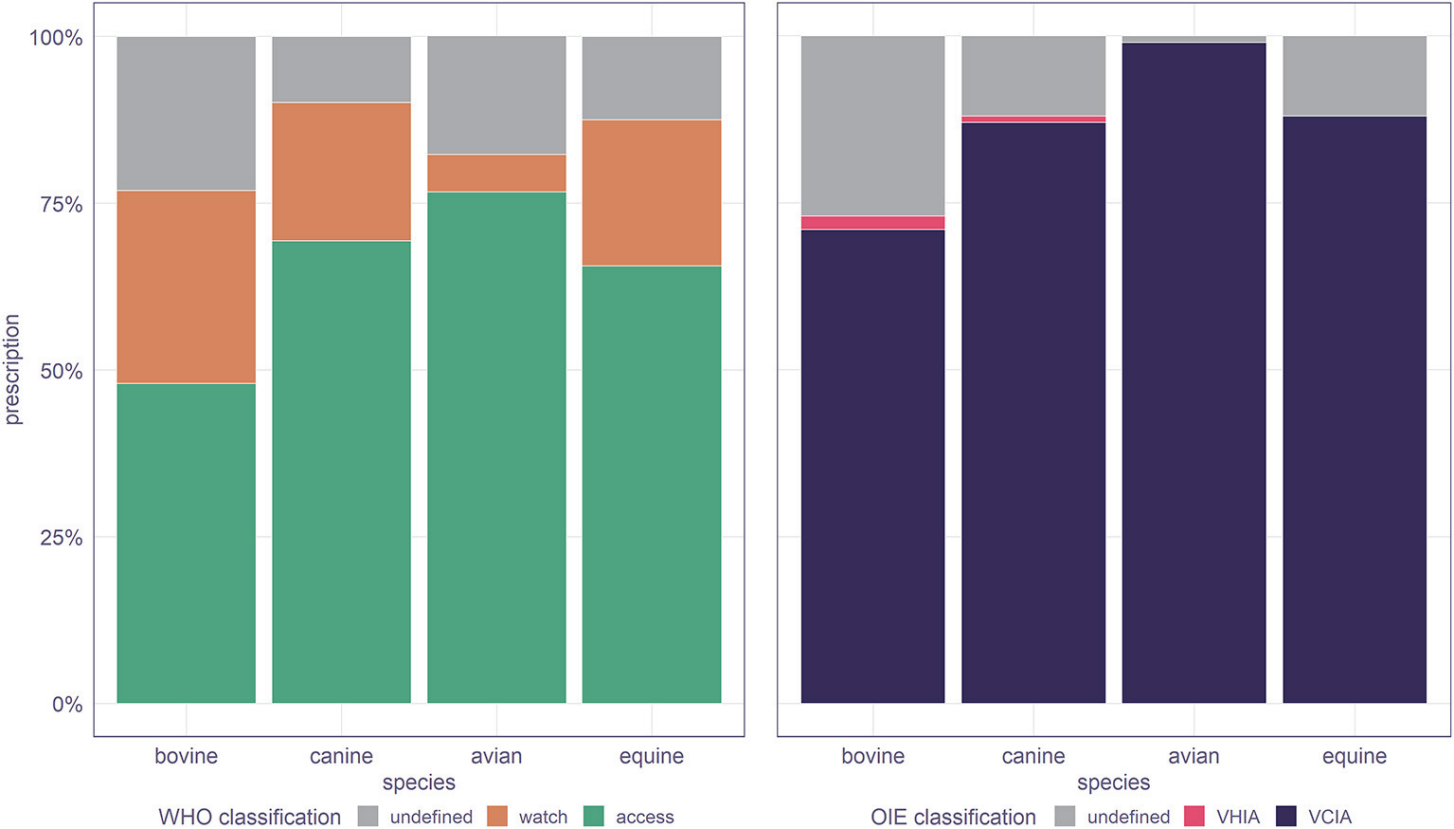
Antibiotics prescribed to different species in veterinary consultation records of the WRLDj of Bhutan in 2017, stratified according to the *AWaRe index* and OIE classification. VCIA, veterinary critically important antimicrobial agents; VHIA, veterinary highly important antimicrobial agents.

Accounting for all antibiotic prescription to the top species, between 45% (ruminants) and 75% (avian) corresponded to access agents, between 5% and up to 25% to watch agents, and up to 20% of the antibiotics prescribed had “undefined” classification. As per the OIE guidelines, over 70% of the prescriptions for any species corresponded to VCIA; the prescription of VHIA was limited across species, and about 25% prescriptions had no classification in the list (Figure 5).

## Discussion

Our estimate of the proportion of veterinary consultations that resulted in a prescription of antibiotics is possibly the first for Bhutan, and one of few estimates for LMIC, where the use of antibiotics remains largely undocumented (20). We estimated that a third of all veterinary consultations result in an antibiotic prescription, which suggests a conservative therapeutic use of antibiotics that positively contrasts with the extensive prescription of these agents in other countries in the region (21, 22) and even with the prescription of antibiotic in high-income countries, where up to 85% of veterinary consultations may result in an antibiotic treatment (23).

The proportion estimates reported in this study were derived from a fixed-effect model. Because there is a unique set of Standard Treatment Guidelines (7) used by all AHP since 2017, it was reasonable to assume that the proportion of veterinary consultations that result in an antibiotic prescription across AHP stems from a single distribution (17). We presented three estimates based on a sequential aggregation of data which explored and acknowledged the potential variability introduced by the AHP sourcing the data. Overall, the estimates and confidence intervals for the region were consistent across the analysis sets. The proportion of consultations that resulted in antibiotic prescription was particularly similar between the AHPs deemed adequate replacements included in set 2. These AHP are part of a single Dzongkhag and receive technical support from the same DVH (Figure 1), which likely explain the homogeneity observed.

Despite a consistent prescription proportion estimate for the region, the study revealed substantial prescription proportion heterogeneity between the 23 AHP scrutinised (*i*^2^ = 73%). *A priori*, it was hypothesized that the mix of species presented to each AHP could explain the variability, as some species were found to be frequently treated with antibiotics (avian and equine). Detailed quantification of the type of consultation per AHP (Figure 3), however, demonstrated that the species mix is an unlikely determinant of the heterogeneous pattern observed. This analysis, however, does not suffice to investigate the heterogeneity. Variable prescription of antibiotics across AHP may arise from the type and number of animals presented to a single consultation, type of syndromic presentations, practitioner's training and experience, etiologic ascertainment, and interpretation of treatment guidelines, immediate availability of antibiotics, regional clustering, among other factors. Moreover, complex cases that require an antibiotic prescription may be transferred to DVHs, increasing the antibiotic prescription proportion associated with these premises. Because the exploration of these factors is framed by a different research question beyond the scope of this manuscript, we will explore this heterogeneity in a subsequent study.

The Standard Treatment Guidelines of Bhutan (7) lists and describes the multietiologic nature associated with signs and suggests alternative therapeutic options. However, it is up to the practitioner to determine the most adequate treatment approach. Thus, whether an antibiotic prescription was appropriate to treat diarrhea, wounds, eye infection, anorexia, and other signs described in consultation records should be evaluated case by case. To assess antibiotic stewardship more accurately, it would be critical that consultation records better document clinical investigations, the use of diagnostic tests, and the outcome of antibiotic courses prescribed. Having these caveats into consideration, the two top diagnoses for which antibiotics are the first therapeutic option in the Standard Treatment Guidelines—bovine mastitis and equine strangles—were treated as per the guideline's recommendations.

As part of our *post-hoc* analyses, we reported the incidence of antibiotic prescription per species stratified by AHP. The analysis showed that the prescription to avian species is frequent and markedly higher in consultations dispensed by LEC. The prescription was primarily tetracyclines (tetracycline HCL powder) to treat diarrhea, which is not a pathognomonic sign of bacterial disease as it can be caused by bacterial (e.g., Fowl cholera) or viral (e.g., avian influenza) infection, parasitic infestation (e.g., coccidiosis, worms), climate (heat stress), or normal physiological processes that could be misdiagnosed (cecal droppings) (24). While the Standard Treatment Guidelines recommend the prescription of this agent to treat fowl cholera (*Pasteurella multocida*) and fowl typhoid (*Salmonella gallinarum*) on a dose of 1 g/L of drinking water for 7 days (7), the agent is not recommended to treat birds producing eggs for human consumption as pharmacokinetics studies have shown that half of this dose administered for 5 days may result in detectable residues of tetracycline in the yolk for up to 9 days after the treatment ceased (25). Understanding and standardising the use of antibiotics in this species is a priority of the National Action Plan on Antimicrobial Resistance (26), and there are ongoing efforts to study and improve prescription in this sector. Eggs are one of two foods of animal origin widely produced and consumed in Bhutan (the other being milk) which are compatible with the country's Buddhist principles of animal compassion (27). Given the importance of eggs in the Bhutanese diet, the use of tetracyclines and other antibiotic agents in layers requires close monitoring.

Another aspect of antibiotic stewardship informed by this study is the type of antibiotic prescribed as per the WHO's *AWaRe index*. Although the preferred prescription usually corresponds to an *access* agent, the ample use of *watch* antibiotics to treat common health issues in bovine, particularly mastitis and teat cracks, requires close surveillance for three reasons: (a) the importance of these antibiotics for human health; (b) the importance of milk from a nutritional and economic perspective in this country; and (c) because farmers may not be familiar with the concept of withdrawal periods. The intramammary formulations and injectable oxytetracycline used to treat ruminants have withdrawal periods of at least 96 h in large ruminants and 72 h in small ones (28); nonetheless, it is unclear if this is informed by the practitioners and practiced by the farmers. Although in-country initiatives (8, 26) have resulted in a permanent evaluation of antibiotic classes being used in food-producing animals, Bhutan will remain a small veterinary pharmaceutical market challenged to prioritise the purchase of some high relevance antibiotics to ensure both the optimal use of public funding and that all animal health needs are covered. In this context, the AWaRe index seems a more informative approach than the OIE classification to track the progress of initiatives seeking to improve antibiotic use, evaluate interventions, and define antibiotic stewardship goals (26). As only 7% of all medicinal products registered in Bhutan are veterinary allopathic agents (29), the economic-veterinary-public-health trade-off is of One Health importance and requires that the prescription of antibiotics not only to well-established dairy and poultry systems but also to other emerging production sectors that already face infectious disease challenges (30) is followed closely.

This study used handwritten veterinary records that were manually digitalised for analysis. The process is unlikely to have introduced significant bias because all veterinary records in Bhutan are written in English, the terminology employed by the veterinary service was consistent across premises, and the transcription was assisted by LEC and DVH personnel when needed. Notwithstanding, many records had a range of inputs missing, and many AHP had less than expected consultation records available, which may have reduced the precision of our estimates. To account for these pitfalls, we assumed in the study design stage a high intracluster correlation (0.04) which boosted the number of clusters needed to estimate the underlying proportion investigated. The results produced by the study seem robust: the prescribing proportion estimates derived from the three sets of data were consistent, the ICC for the most extensive dataset (set 3) was smaller than initially estimated (~0.03), and more clusters than needed were available for inclusion in the study (23 instead of 17).

We envision three uses for the results in this study. First, the study provides a benchmark to compare the progress made around antibiotic stewardship in Bhutan. Second, the study provides a methodology to investigate the proportion of veterinary consultations that result in an antibiotic prescription across Bhutan and other nations with a similar veterinary service structure. Third, the study highlights areas that can be enhanced toward improved antibiotic stewardship. These areas include the revision of antibiotic recommendations for certain species, close monitoring of watch antibiotics prescribed to key livestock species and ascertainment of withdrawal periods, and improved consultation records, including clinical investigation, diagnostic test, and treatment outcome. Considering the limitations previously described, our results suggest that the Bhutanese veterinary service practices a conservative prescription of antibiotics in terms of frequency and type as per the AWaRe index, which is a positive feature of antimicrobial stewardship. The Bhutanese approach to antibiotic prescription, which is managed by a hierarchical veterinary service and harmonised through standard treatment guidelines, may serve as an example, and could help in guiding veterinary antimicrobial stewardship processes in other low- and middle-income countries.

## Data Availability Statement

The datasets presented in this article are not readily available because the datasets contain identifiable data of people. Requests to access the datasets should be directed to jp.villanueva@unimelb.edu.au.

## Author Contributions

JV-C, MS, and JM conceptualized the study design, sample size, and fieldwork with input from KR, SD, UN, PT, and PS. JV-C carried out the fieldwork with collaboration from KR, SD, UN, PT, and PS. JV-C undertook all analyses with input from MS and JM. JV-C wrote the main manuscript text. All authors were involved in the design of the study, reviewed and commented on the manuscript.

## Conflict of Interest

The authors declare that the research was conducted in the absence of any commercial or financial relationships that could be construed as a potential conflict of interest.

## Supplementary Material

Supplementary Figure 1 Top 5 diagnosis most frequently treated with antibiotics and associated antibiotic class prescription. Antibiotics classes are colored according to the *AWaRe index*; green: *access*; amber: *watch*; gray*: unclassified*. ^*^and ^∧^ were separated from their classes as these drugs have different classification. Unsp. wound, unspecific wound; miscellaneous, unspecific signs including swollen, stress, and unconnected terms including “preventive” and “no diagnosis”.

Supplementary Figure 2 Frequency of diagnoses found in consultation records (treated and not treated with antibiotics) grouped by syndrome or system affected. GI, gastrointestinal afflictions, including diarrhea and vomits; Infectdiseases, infectious diseases including strangles and FMD; int-ext-parasite, internal or external parasites; other, unspecific signs including swollen, sore, or prolapse without further specification, poisoning, unconnected terms including “preventive,” “no diagnosis,” and illegible records; systemic, weakness and inactivity, anorexia, or ill-thrift.

Supplementary Figure 3 Frequency of prescription of antibiotic classes in the consultation records scrutinized.
