# Supplementary figures and images for: Antibiotic Prescription in Veterinary Consultations in Bhutan: A Retrospective Cross-Sectional Study
Source: Front Vet Sci. 2021 May 28;8:641488. doi: 10.3389/fvets.2021.641488 (PMC8194083; doi:10.3389/fvets.2021.641488)

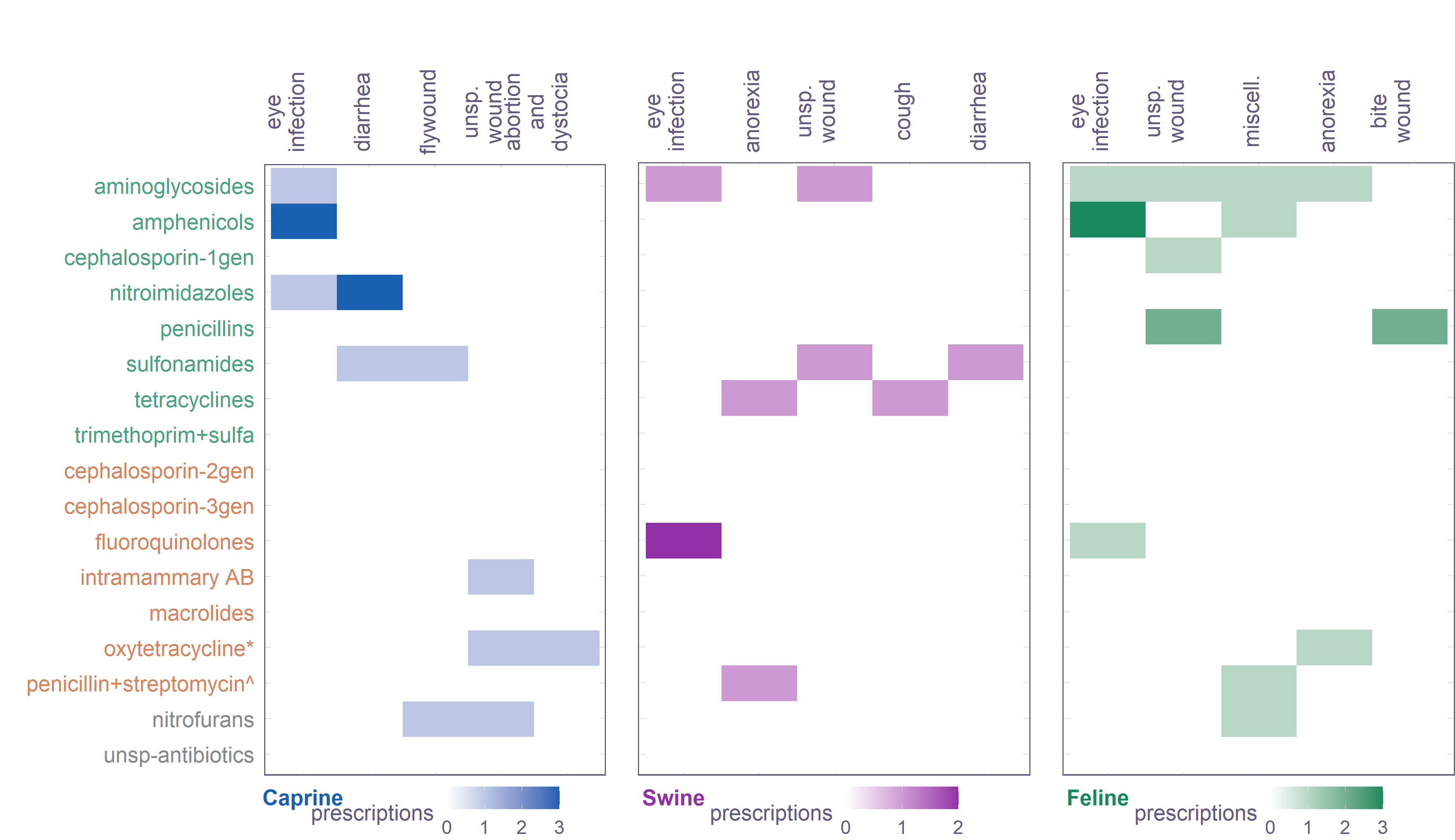

Supplement: Supplementary Figure 1 — Top 5 diagnosis most frequently treated with antibiotics and associated antibiotic class prescription. Antibiotics classes are colored according to the AWaRe index; green: access; amber: watch; gray: unclassified. *and ∧ were separated from their classes as these drugs have different classification. Unsp. wound, unspecific wound; miscellaneous, unspecific signs including swollen, stress, and unconnected terms including “preventive” and “no diagnosis”. [file Image_1.TIFF]

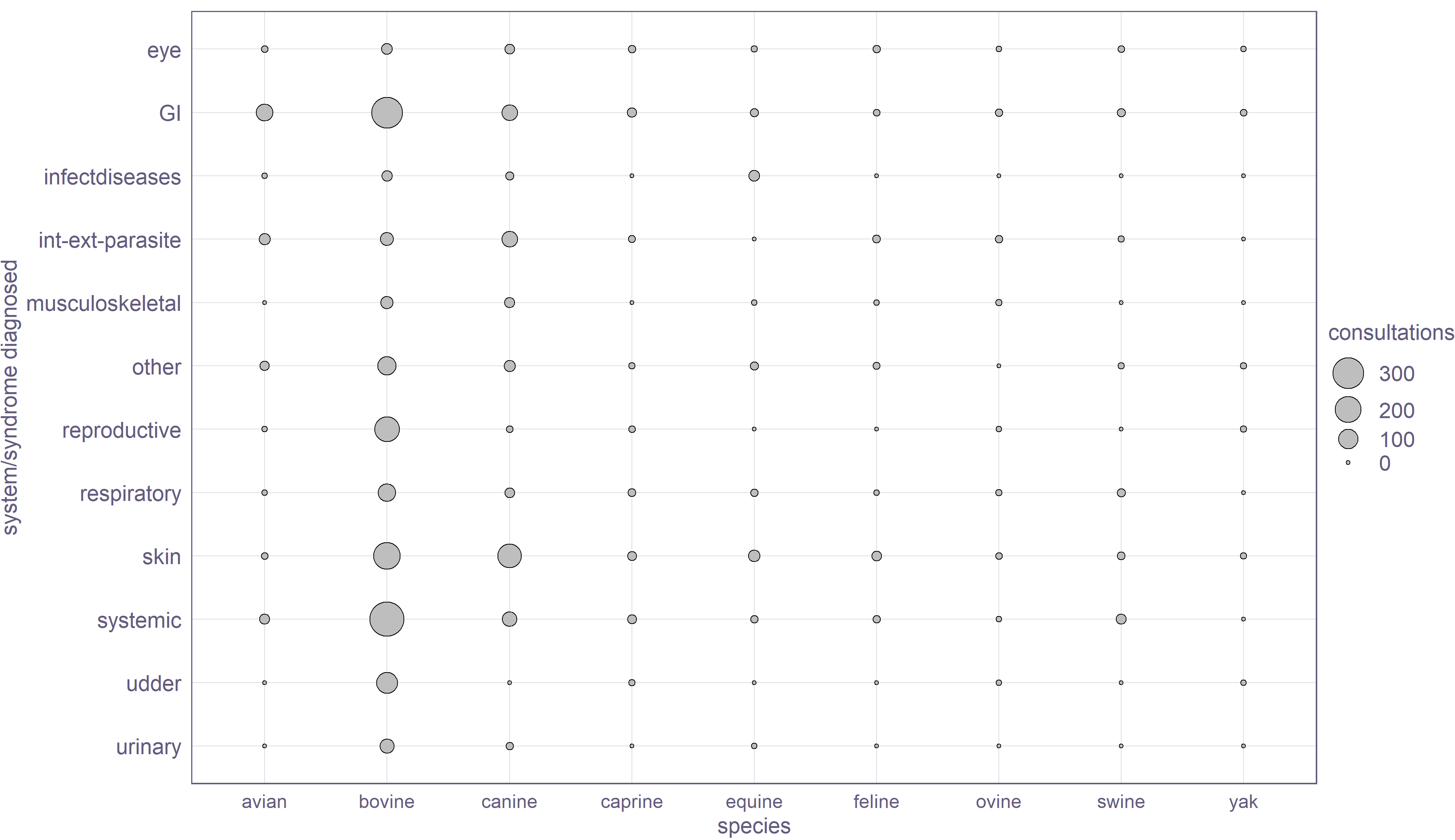

Supplement: Supplementary Figure 2 — Frequency of diagnoses found in consultation records (treated and not treated with antibiotics) grouped by syndrome or system affected. GI, gastrointestinal afflictions, including diarrhea and vomits; Infectdiseases, infectious diseases including strangles and FMD; int-ext-parasite, internal or external parasites; other, unspecific signs including swollen, sore, or prolapse without further specification, poisoning, unconnected terms including “preventive,” “no diagnosis,” and illegible records; systemic, weakness and inactivity, anorexia, or ill-thrift. [file Image_2.TIFF]

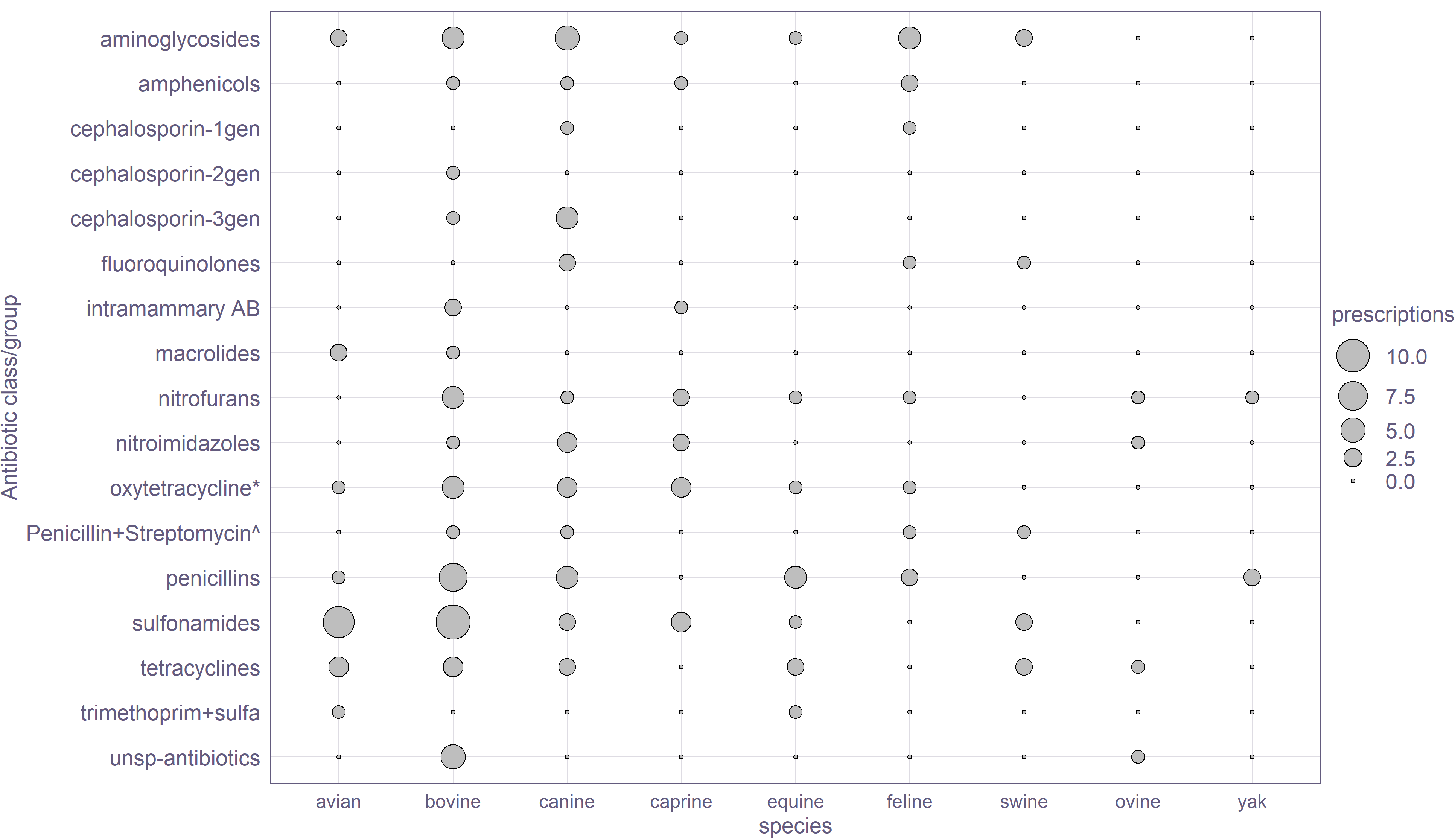

Supplement: Supplementary Figure 3 — Frequency of prescription of antibiotic classes in the consultation records scrutinized. [file Image_3.TIFF]
